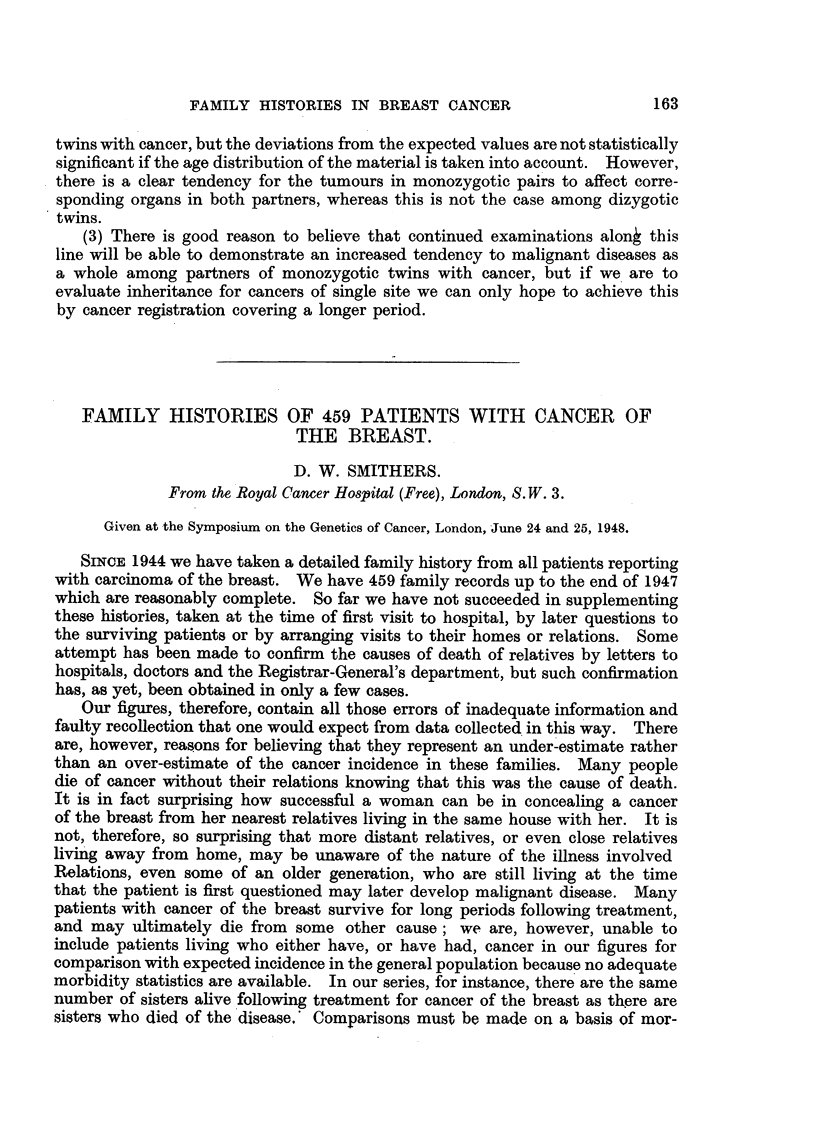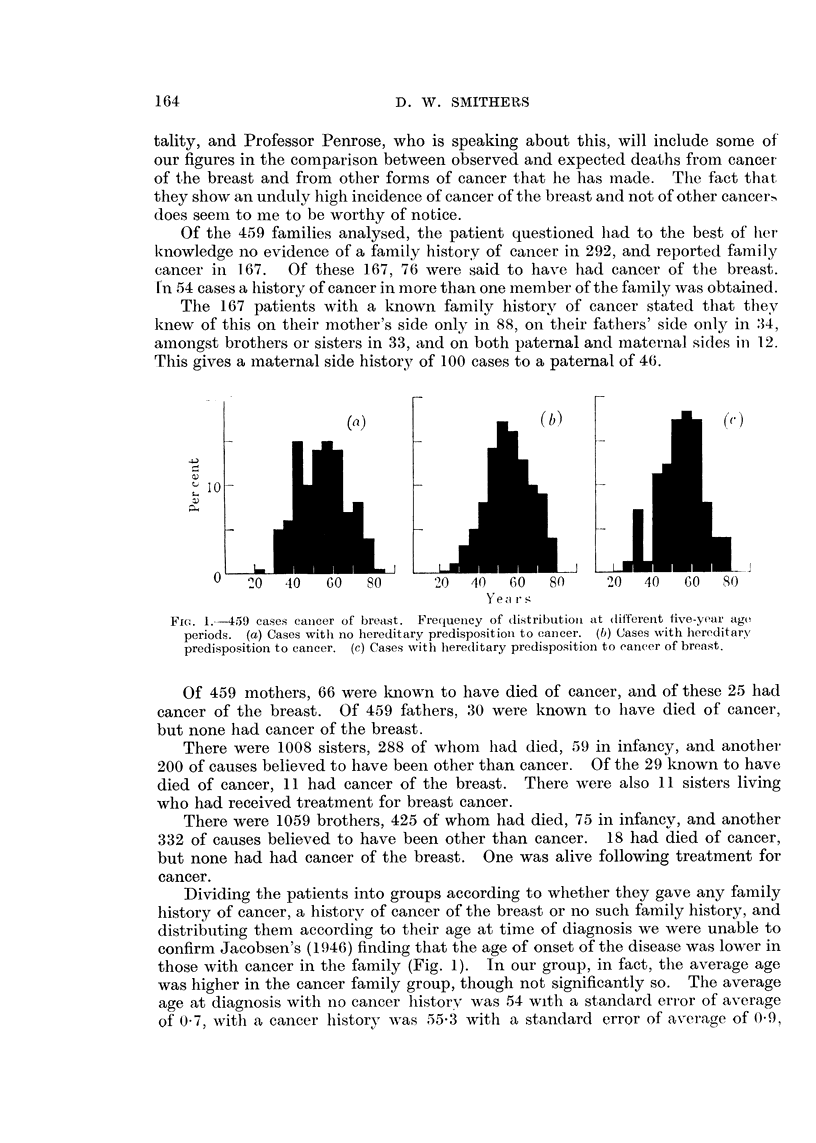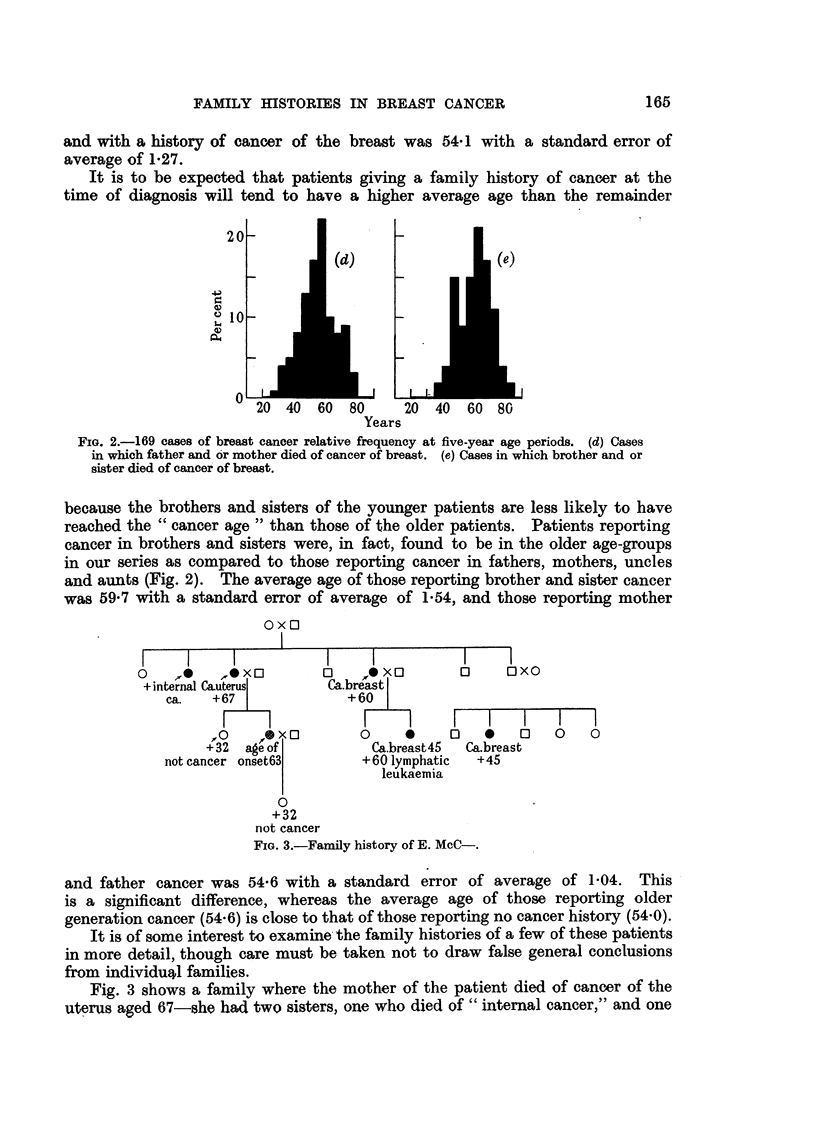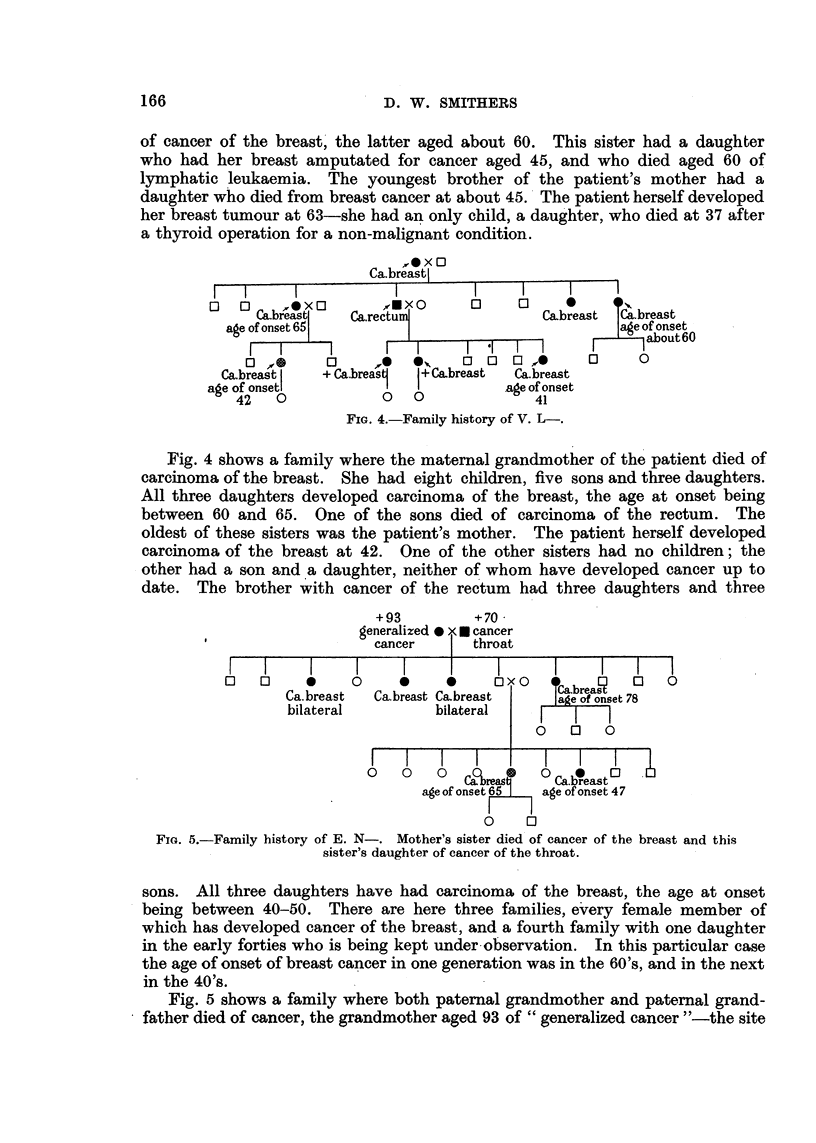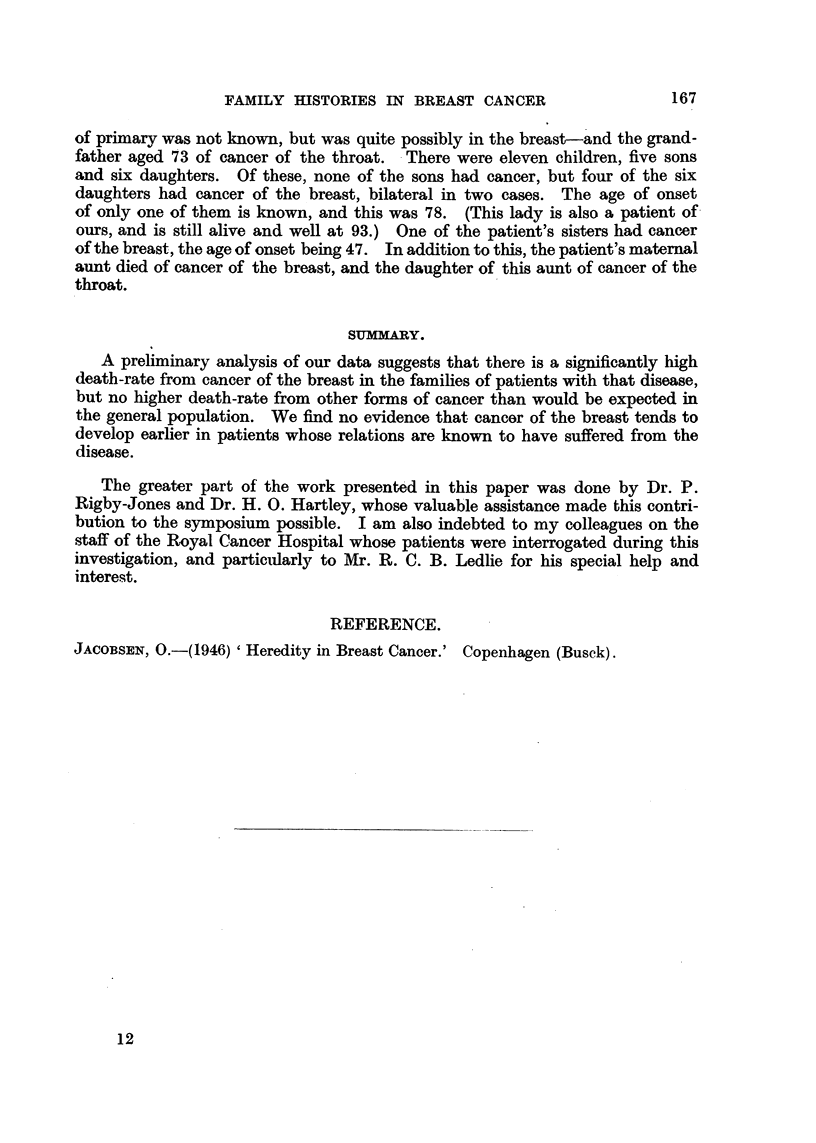# Family Histories of 459 Patients with Cancer of the Breast

**DOI:** 10.1038/bjc.1948.24

**Published:** 1948-06

**Authors:** D. W. Smithers


### FAMILY HISTORIES OF 459 PATIENTS WITH CANCER OF THE BREAST.

D. W. SMITHERS.

*From the Royal Cancer Hospital (Free), London, S.W. 3.*

Given at the Symposium on the Genetics of Cancer, London, June 24 and 25, 1948.

SINCE 1944 we have taken a detailed family history from all patients reporting with carcinoma of the breast. We have 459 family records up to the end of 1947 which are reasonably complete. So far we have not succeeded in supplementing these histories, taken at the time of first visit to hospital, by later questions to the surviving patients or by arranging visits to their homes or relations. Some attempt has been made to confirm the causes of death of relatives by letters to hospitals, doctors and the Registrar-General's department, but such confirmation has, as yet, been obtained in only a few cases.

Our figures, therefore, contain all those errors of inadequate information and faulty recollection that one would expect from data collected in this way. There are, however, reasons for believing that they represent an under-estimate rather than an over-estimate of the cancer incidence in these families. Many people die of cancer without their relations knowing that this was the cause of death. It is in fact surprising how successful a woman can be in concealing a cancer of the breast from her nearest relatives living in the same house with her. It is not, therefore, so surprising that more distant relatives, or even close relatives living away from home, may be unaware of the nature of the illness involved Relations, even some of an older generation, who are still living at the time that the patient is first questioned may later develop malignant disease. Many patients with cancer of the breast survive for long periods following treatment, and may ultimately die from some other cause ; we are, however, unable to include patients living who either have, or have had, cancer in our figures for comparison with expected incidence in the general population because no adequate morbidity statistics are available. In our series, for instance, there are the same number of sisters alive following treatment for cancer of the breast as there are sisters who died of the disease. Comparisons must be made on a basis of mortality, and Professor Penrose, who is speaking about this, will include some of our figures in the comparison between observed and expected deaths from cancer of the breast and from other forms of cancer that he has made. The fact that they show an unduly high incidence of cancer of the breast and not of other cancers does seem to me to be worthy of notice.

Of the 459 families analysed, the patient questioned had to the best of her knowledge no evidence of a family history of cancer in 292, and reported family cancer in 167. Of these 167, 76 were said to have had cancer of the breast. In 54 cases a history of cancer in more than one member of the family was obtained.

The 167 patients with a known family history of cancer stated that they knew of this on their mother's side only in 88, on their fathers' side only in 34, amongst brothers or sisters in 33, and on both paternal and maternal sides in 12. This gives a maternal side history of 100 cases to a paternal of 46.

FIG. 1.—459 cases cancer of breast. Frequency of distribution at different five-year age periods. (a) Cases with no hereditary predisposition to cancer. (b) Cases with hereditary predisposition to cancer. (c) Cases with hereditary predisposition to cancer of breast.

Of 459 mothers, 66 were known to have died of cancer, and of these 25 had cancer of the breast. Of 459 fathers, 30 were known to have died of cancer, but none had cancer of the breast.

There were 1008 sisters, 288 of whom had died, 59 in infancy, and another 200 of causes believed to have been other than cancer. Of the 29 known to have died of cancer, 11 had cancer of the breast. There were also 11 sisters living who had received treatment for breast cancer.

There were 1059 brothers, 425 of whom had died, 75 in infancy, and another 332 of causes believed to have been other than cancer. 18 had died of cancer, but none had had cancer of the breast. One was alive following treatment for cancer.

Dividing the patients into groups according to whether they gave any family history of cancer, a history of cancer of the breast or no such family history, and distributing them according to their age at time of diagnosis we were unable to confirm Jacobsen's (1946) finding that the age of onset of the disease was lower in those with cancer in the family (Fig. 1). In our group, in fact, the average age was higher in the cancer family group, though not significantly so. The average age at diagnosis with no cancer history was 54 with a standard error of average of 0·7, with a cancer history was 55·3 with a standard error of average of 0·9, and with a history of cancer of the breast was 54·1 with a standard error of average of 1·27.

It is to be expected that patients giving a family history of cancer at the time of diagnosis will tend to have a higher average age than the remainder

FIG. 2.—169 cases of breast cancer relative frequency at five-year age periods. (*d*) Cases in which father and or mother died of cancer of breast. (*e*) Cases in which brother and or sister died of cancer of breast.

because the brothers and sisters of the younger patients are less likely to have reached the " cancer age " than those of the older patients. Patients reporting cancer in brothers and sisters were, in fact, found to be in the older age-groups in our series as compared to those reporting cancer in fathers, mothers, uncles and aunts (Fig. 2). The average age of those reporting brother and sister cancer was 59·7 with a standard error of average of 1·54, and those reporting mother

FIG. 3.—Family history of E. McC—.

and father cancer was 54·6 with a standard error of average of 1·04. This is a significant difference, whereas the average age of those reporting older generation cancer (54·6) is close to that of those reporting no cancer history (54·0).

It is of some interest to examine the family histories of a few of these patients in more detail, though care must be taken not to draw false general conclusions from individual families.

Fig. 3 shows a family where the mother of the patient died of cancer of the uterus aged 67—she had two sisters, one who died of " internal cancer," and one of cancer of the breast, the latter aged about 60. This sister had a daughter who had her breast amputated for cancer aged 45, and who died aged 60 of lymphatic leukaemia. The youngest brother of the patient's mother had a daughter who died from breast cancer at about 45. The patient herself developed her breast tumour at 63—she had an only child, a daughter, who died at 37 after a thyroid operation for a non-malignant condition.

FIG. 4.—Family history of V. L—.

Fig. 4 shows a family where the maternal grandmother of the patient died of carcinoma of the breast. She had eight children, five sons and three daughters. All three daughters developed carcinoma of the breast, the age at onset being between 60 and 65. One of the sons died of carcinoma of the rectum. The oldest of these sisters was the patient's mother. The patient herself developed carcinoma of the breast at 42. One of the other sisters had no children; the other had a son and a daughter, neither of whom have developed cancer up to date. The brother with cancer of the rectum had three daughters and three

FIG. 5.—Family history of E. N—. Mother's sister died of cancer of the breast and this sister's daughter of cancer of the throat.

sons. All three daughters have had carcinoma of the breast, the age at onset being between 40–50. There are here three families, every female member of which has developed cancer of the breast, and a fourth family with one daughter in the early forties who is being kept under observation. In this particular case the age of onset of breast cancer in one generation was in the 60's, and in the next in the 40's.

Fig. 5 shows a family where both paternal grandmother and paternal grandfather died of cancer, the grandmother aged 93 of " generalized cancer "—the site of primary was not known, but was quite possibly in the breast—and the grandfather aged 73 of cancer of the throat. There were eleven children, five sons and six daughters. Of these, none of the sons had cancer, but four of the six daughters had cancer of the breast, bilateral in two cases. The age of onset of only one of them is known, and this was 78. (This lady is also a patient of ours, and is still alive and well at 93.) One of the patient's sisters had cancer of the breast, the age of onset being 47. In addition to this, the patient's maternal aunt died of cancer of the breast, and the daughter of this aunt of cancer of the throat.

### SUMMARY.

A preliminary analysis of our data suggests that there is a significantly high death-rate from cancer of the breast in the families of patients with that disease, but no higher death-rate from other forms of cancer than would be expected in the general population. We find no evidence that cancer of the breast tends to develop earlier in patients whose relations are known to have suffered from the disease.

The greater part of the work presented in this paper was done by Dr. P. Rigby-Jones and Dr. H. O. Hartley, whose valuable assistance made this contribution to the symposium possible. I am also indebted to my colleagues on the staff of the Royal Cancer Hospital whose patients were interrogated during this investigation, and particularly to Mr. R. C. B. Ledlie for his special help and interest.

### REFERENCE.

JACOBSEN, O.—(1946) 'Heredity in Breast Cancer.' Copenhagen (Busck).